# A Bodyweight-Dependent Allometric Exponent for Scaling Clearance Across the Human Life-Span

**DOI:** 10.1007/s11095-012-0668-x

**Published:** 2012-01-28

**Authors:** Chenguang Wang, Mariska Y. M. Peeters, Karel Allegaert, Heleen J. Blussé van Oud-Alblas, Elke H. J. Krekels, Dick Tibboel, Meindert Danhof, Catherijne A. J. Knibbe

**Affiliations:** 1 Division of Pharmacology, LACDR, Leiden University, Leiden, the Netherlands; 2 Intensive Care and Department of Paediatric Surgery, Erasmus MC Sophia Children’s Hospital, Rotterdam, the Netherlands; 3 Department of Clinical Pharmacy, St. Antonius Hospital, P.O. Box 2500, 3430 EM Nieuwegein, the Netherlands; 4 Neonatal Intensive Care Unit, University Hospitals Leuven, Leuven, Belgium; 5 Department of Anesthesiology, Erasmus Medical Center, Rotterdam, the Netherlands

**Keywords:** allometric, clearance, life-span, pharmacokinetics, propofol

## Abstract

**Purpose:**

To explore different allometric equations for scaling clearance across the human life-span using propofol as a model drug.

**Methods:**

Data from seven previously published propofol studies ((pre)term neonates, infants, toddlers, children, adolescents and adults) were analysed using NONMEM VI. To scale clearance, a bodyweight-based exponential equation with four different structures for the exponent was used: (I) 3/4 allometric scaling model; (II) mixture model; (III) bodyweight-cut-point separated model; (IV) bodyweight-dependent exponent model.

**Results:**

Model I adequately described clearance in adults and older children, but overestimated clearance of neonates and underestimated clearance of infants. Use of two different exponents in Model II and Model III showed significantly improved performance, but yielded ambiguities on the boundaries of the two subpopulations. This discontinuity was overcome in Model IV, in which the exponent changed sigmoidally from 1.35 at a hypothetical bodyweight of 0 kg to a value of 0.56 from 10 kg onwards, thereby describing clearance of all individuals best.

**Conclusions:**

A model was developed for scaling clearance over the entire human life-span with a single continuous equation, in which the exponent of the bodyweight-based exponential equation varied with bodyweight.

## INTRODUCTION

For scaling pharmacokinetics across the human life-span, the 3/4 allometric scaling approach (1) has gained in popularity in the field of pediatrics. While the 3/4 allometric scaling approach was originally designed to describe metabolic rates between different species covering a wide range in bodyweight (2), this function is now being applied to parameterize the influence of changes in body size on drug clearance parameters within the human weight-range.

In contrast with the reports supporting the 3/4 allometric scaling principles (3–5), there is a number of theoretical arguments against these scaling principles in general (6–8). Furthermore, the value of the allometric exponent is debated (9,10), the existence of a unique and universal value for the exponent is thought to be unlikely (11,12) and the application of allometric scaling principles to pharmacokinetics in the human weight-range is disputed (13,14). For paediatrics, the covering of changes in body size made by this bodyweight-based allometric equation with a fixed exponent of 0.75 for clearance are specifically insufficient to describe and predict drug clearance in preterm and term neonates, infants and young children (13,15,16). When applying the 3/4 allometric scaling method to different drugs, it has been found that the model systematically over-predicted clearances for neonates and under-predicted clearances for infants (15,17).

In order to account for the discrepancy of the 3/4 allometric method in young children, an augmentation of the 3/4 allometric equation with an age-based sigmoidal equation has been proposed (18,19). However, this age-adjustment is unidirectional, whereas bi-directional changes from the 3/4 allometric line have been reported in the papers aforementioned, that depend on the age of the children (15,17). Furthermore, introduction of this age-based maturation equation introduces age into the model in addition to bodyweight. While age and bodyweight are highly correlated in the paediatric population in a nonlinear manner, this may result in a collinearity problem (20). In this respect, it is emphasized that recent research shows that *a priori* inclusion of a false predefined covariate relationship into a model may decrease the predictive performance of that model (21). As a result of these limitations, in some reports the exponent of the allometric scaling equation was estimated instead of fixed to 0.75, resulting in values higher than 1 in datasets of young infants (22,23). From these reports, it seems that an optimized and statistically sound scaling approach is needed for scaling of clearance in paediatrics.

The aim of this study was to explore new approaches to scale drug clearance over the entire human life-span. Propofol was used as a model drug, and for this study seven datasets were available from preterm neonates with a median (range) gestational age of 37 (26–40) weeks to 81 year old adults (15,24–29).

## MATERIALS AND METHODS

### Subjects

A total of 174 subjects from seven previously published propofol studies were included in the current study. These studies are described in detail elsewhere and are briefly discussed as relevant to the current analysis.

#### Neonates (24)

Twenty-five cardiovascularly and respiratory stable neonates with a median of bodyweight of 2.93 (range 0.68–4.03) kilograms, postnatal age of 8 (1–25) days and gestational age of 37 (26–40) weeks were given an intravenous bolus dose of propofol (3 mg⋅kg^−1^) for the elective removal of chest tubes, (semi)elective chest tube placement or endotracheal intubation.

#### Infants (25)

Twenty-two non-ventilated infants after major craniofacial surgery with a median bodyweight of 8.9 (4.8–12.5) kilograms, aged 10 (3.8–17.3) months received 2–4 mg⋅kg^−1^·h^−1^ propofol during a median of 12.5 (6.0–18.1) hours.

#### Toddlers (26)

Twelve toddlers with minor burns, who had a median bodyweight of 11.2 (8.7–18.9) kilograms and age of 17.8 (12–31) months, were administered 4 mg⋅kg^−1^ propofol just before bathing.

#### Children (27)

Fifty-three healthy unpremedicated children with a median bodyweight of 23.3 (15–60.5) kilograms and median age of 7 (3–11) years were studied. Twenty children received an intravenous loading dose of 3 mg⋅kg^−1^ propofol. In the remaining 33 children, an intravenous loading dose of 3.5 mg⋅kg^−1^ was followed by a maintenance infusion. In 18 of the 33 children, a single infusion rate of 0.15 mg⋅kg^−1^⋅min^−1^ was administered, while 15 children received an infusion of 0.20 mg⋅kg^−1^⋅min^−1^ for 30 min, followed by an infusion of 0.125 mg⋅kg^−1^⋅min^−1^ till the end of the procedure.

#### Adolescents (15)

Fourteen adolescents with a median bodyweight of 51 (36.6–82) kilograms and median age of 14.7 (9.8–20.1) years were anaesthetized with propofol-remifentanil (2–10 mg⋅kg^−1^·h^−1^) for scoliosis surgery during 6.8 (3.3–7.7) hours with an intra-operative wake-up test followed by re-induction of anesthesia.

#### Adults I (28)

Twenty-four women undergoing gynaecological surgery, with a median bodyweight of 68.5 (55–80) kilograms and a median age of 45.5 (33–57) years, received 2.5 mg⋅kg^−1^ propofol over 60 s for induction of anesthesia.

#### Adults II (29)

Twenty-four healthy volunteers with a median bodyweight of 79.4 (44.4–122.7) kilograms and median age of 53 (26–81) years were administered a bolus dose of propofol, followed 1 h later by a 60 min infusion with an infusion rate of 25, 50, 100, or 200 mg⋅kg^−1^·min^−1^ in a study which investigated the influences of method of administration, infusion rate, patient covariates, and EDTA (ethylenediaminetetraacetic acid) on the pharmacokinetics of propofol.

### Pharmacokinetic Modeling

#### Model Building

The population pharmacokinetic analysis was performed with the non-linear mixed effects modeling software NONMEM version 6.2. (ICON Development Solutions, Ellicott City, MD, USA) using the first-order conditional estimation method with the interaction option (FOCEI). Tools like S-PLUS interface for NONMEM (LAP&P Consultants BV, Leiden, NL), S-Plus (version 8.1, Insightful Software, Seattle, WA, USA), XPose and R (version 2.10.0) were used to visualize the output and evaluate the models.

Propofol concentrations were logarithmically transformed and fitted simultaneously, since the range in concentrations was more than 1,000 fold. Model building was performed in four steps: (1) selection of structural model, (2) selection of statistical sub-model, (3) covariate analysis, (4) model validation. A difference in objective function (OFV) between models of more than 7.88 points was considered as statistically significant (*p* < 0.005 assuming a Chi-square distribution). Furthermore, the goodness-of-fit plots (observed *versus* individual predicted concentrations and *versus* population predicted concentrations, and conditional weighted residuals *versus* time and *versus* population prediction concentrations) were evaluated (30). Finally, improvement of the individual concentration-time profiles, the confidence intervals of the parameter estimates and the correlation matrix were assessed.

#### Structural Model

Based on previous reports (31–33) the time-course of propofol concentrations was modeled with a three-compartment model, which was parameterized in terms of total clearance (CL), volume of distribution of the central compartment (V1), volume of distribution of the rapid-equilibrating peripheral compartment (V2) and slow-equilibrating peripheral compartment (V3), and inter-compartmental clearances between central compartment and two peripheral compartments (Q2,Q3). In addition, the performance of a two compartment model with the parameters CL, Q2, V1 and V2 was evaluated.

#### Statistical Model

Inter-individual variability in the pharmacokinetic parameters was tested in the model assuming log-normal distributions, expressed as

1 $$ {\theta_i} = {\theta_{TV}} \times {e^{{\eta_i}}},\;{\eta_i}\sim N(0,{\omega^2}) $$

where *θ*_*i*_ is the individual pharmacokinetic parameter value for the *i*th individual, *θ*_*TV*_ is the population pharmacokinetic parameter value or typical value, and *η*_*i*_ is a random variable for the *i*th individual from a normal distribution with mean zero and variance *ω* ^2^. In addition to testing the inclusion of inter-individual variability on individual parameters, model improvement by inclusion of covariance between these variability parameters was tested as well.

For the residual error, an additive model for log-transformed concentrations was used which corresponds to proportional error on untransformed data, expressed as:

2 $$ \log {C_{ij}} = \log {C_{pred}}_{ij} + \varepsilon, \;\varepsilon \sim N(0,{\sigma^2}) $$

where *C*_*ij*_ is the value of the observed propofol concentration of *i*th individual at time *j*, ${C_{pre{d_{ij}}}}$ is the value of the predicted propofol concentration of the *i*th individual at time *j*, and *ε* is a random variable for this observation from a normal distribution with mean zero and variance *σ* ^2^.

#### Covariate Models

To visualize potential relationships, candidate covariates such as age and bodyweight were plotted independently *versus* the empirical Bayes *post hoc* estimates for all pharmacokinetic parameters. Potential covariates were separately implemented into the model using a linear or power equation over the studied covariate range:

3 $$ {\theta_i} = {\theta_{TV}} \bullet {(\frac{{Cov}}{{Co{v_{Median}}}})^k} $$

In this equation, *θ*_*i*_ represents the individual parameter estimate of the *i*th subject, *θ*_*TV*_ represents the population parameter estimate, *Cov* is the covariate of interest with subscript *median* indicating the median value of the particular covariate and *k* is the exponent which was fixed to 1 for a linear function or estimated for a power function.

The significance of a covariate was statistically tested by use of the objective function. A *p* value < 0.005 was considered significant in the forward inclusion while on the other hand a more stringent *p* value of < 0.001 was used in the backward deletion. In addition, individual and population parameter estimates were plotted against the most predictive covariate to evaluate whether the individual predicted parameters are evenly distributed around the population predicted values (30). When two or more covariates were found to significantly improve the model, the covariate causing the largest reduction in the objective function was retained in the model and served as the basis for subsequent inclusion of additional covariates. Furthermore, for the final covariate model, general criteria for model evaluation were considered as described under ‘Model Building’, while also the results of the model validation procedures were taken into account.

As bodyweight proved to be superior over age as a covariate for clearance, four covariate models based on bodyweight were tested. For propofol clearance, the common structure of these allometric models was:

4 $$ C{l_i} = C{l_p} \times {\left( {\frac{{B{W_i}}}{{70}}} \right)^k} $$

in which *Cl*_*i*_ is clearance in the *i*th individual with bodyweight *BW*_*i*_; *Cl*_*p*_ is the clearance in a standardized adult with a bodyweight of 70 kg; and the exponent *k* has different values in the four different covariate models as described below.

##### Model I.

The 3/4 allometric model; in which *k* was fixed to 0.75.

##### Model II.

The mixture model; in which different values for the exponent *k* were estimated for two sub-populations. The entire population was assumed to have two subpopulations: *POP*_*1*_ and *POP*_*2*_. For each sub-population different *k* and *Cl*_*p*_ values were estimated, but the inter-individual variability on *Cl* was kept the same for both sub-populations. The assignment of individuals to one of the sub-populations was determined by a probability model (34). Briefly, the probability model assumed two log-normal distributions for clearance with different mean values but common standard deviation for two sub-populations. Each individual has a probability of *π* belonging to *POP*_*1*_ and has 1-*π* probability of belonging to *POP*_*2*_.The mixture model was implemented by the $MIX option in NONMEM VI.

##### Model III.

The bodyweight-cut-point separated model; in which different values for the exponent *k* were estimated for two sub-populations: *POP*_*bw<d*_ and *POP*_*bw≥d*_. These sub-populations were determined by a cut-point bodyweight *d* which is a fixed effect parameter in NONMEM: individuals with bodyweight less than the cut-point belonged to *POP*_*bw<d*_ and individuals with bodyweight greater than or equal to the cut-point belonged to *POP*_*bw≥d*_.

##### Model IV.

The bodyweight-dependent exponent (BDE) model; in which the allometric exponent *k* changed in a sigmoidal fashion with bodyweight according to Eq. 5:

5 $$ k = {k_0} - \frac{{{k_{\max }} \times B{W_i}^\gamma }}{{k_{50}^\gamma + B{W_i}^\gamma }} $$

in which *BW*_*i*_ is bodyweight of an individual *i*; *k*_*0*_ is the value of the exponent at a theoretical bodyweight of 0 kg; *k*_*max*_ is the maximum decrease of the exponent; *k*_*50*_ is the bodyweight at which a 50% decrease in the maximum decrease of exponent is attained, and *γ* is the Hill coefficient determining the steepness of sigmoidal decline in the exponent.

### Model Validation

Stratified bootstrap re-sampling was performed to obtain the same numbers of patients of each of the seven study populations in the new re-sampled datasets compared to the original dataset. This stratified bootstrap was implemented by the “bootstrap” command and the “-stratify_on” option in Perl-speaks-NONMEM (copyright by Mats Karlsson, Niclas Jonsson and Andrew Hooker). The median, standard deviation and the 90% confidence interval of the parameter estimates were calculated based on the results of the runs that minimized successfully.

In addition, the normalized prediction distribution errors (NPDE) method (35) was used as a simulation-based diagnostic. The dataset was simulated 2,000 times in NONMEM, each observed concentration was subsequently compared to the simulated reference distribution using the NPDE add-on package in R. A histogram of the NPDE distribution in the total dataset and plots of NPDE *versus* individual predicted concentration and *versus* time were used to evaluate the final model.

In order to evaluate the precision of the population predicted clearances by the four different covariate models for clearance, we used the root mean square error (RMSE) which was calculated as follows:

6 $$ RMSE = \sqrt {{\frac{{\sum\nolimits_n {{{(C{l_p} - C{l_i})}^2}} }}{n}}} $$

7 $$ \% RMSE = \frac{{RMSE \times 100}}{{Mean(C{l_i})}} $$

in which *Cl*_*p*_ is the population predicted clearance; *Cl*_*i*_ is the individual predicted clearance; *n* is the number of individuals.

## RESULTS

The analysis was based on a total number of 4,396 observations from 174 individuals aged between 1 day to 81 years and with a bodyweight varying between 0.68 and 122.7 kg. An overview of the datasets used for model building is shown in Table I.

**Table I:** Overview of Propofol Pharmacokinetic Datasets Used in the Current Analysis (Values Expressed as Median (Range))

Population	N	Indication	Weight (kg)	Age (yrs)	Administration	Samples^*c*^
Neonates (24)	25	Removal of chest tubes	2.93(0.68–4.03)	8(1–25)^*a*^	Bolus	4–14
Infants (25)	20	Post-operative sedation after	9(4.8–12.5)	10.2(3.8–17.3)^*b*^	Infusion	4–15
		craniofacial surgery				
Toddlers (26)	12	Bathing of patients with minor burn injuries	11.2(8.74–18.9)	1.25(1–2.6)	Bolus	11–12
Children (27)	53	Superficial body surface surgery	23.3(15–60.5)	7(3–11)	Bolus&infusion	5–18
Adolescents (15)	14	Scoliosis surgery	51(36.6–82)	14.5(9.6–19.8)	Bolus&infusion	6–21
Female adults(28)	24	Gynecological surgery	68.5(55–80)	45.5(33–57)	Bolus	18
Adults (29)	24	PK study in healthy volunteers	79.4(44.4–122.7)	53(26–81)	Infusion	20–21

^*a*^age in days

^*b*^age in months

^*c*^number of samples per subject (range)

A three compartment model performed better than a two compartment model. In this three compartment model, four different bodyweight-based allometric equations for clearance were seperately tested and evaluated for their performance. All parameter estimates including their coefficients of variation (CV values) and objective functions of these four covariate models are listed in Table II.

**Table II:** Parameter Values, Corresponding Coefficients of Variation (CV%), and Objective Function for the Four Different Covariate Models for Clearance. Model I: 3/4 Allometric Model; Model II: the Mixture Model; Model III: the Bodyweight-Cut-Point Separated Model; Model IV: the Bodyweight-Dependent Exponent (BDE) Model

Parameter	Model			
	Model I	Model II	Model III	Model IV
Fixed effect				
Cl (L/min)	= Cl_p_⋅(bw/70)^0.75^	= Cl_p_⋅(bw/70)^k^	= Cl_p_⋅(bw/70)^k^	= Cl_p_⋅(bw/70)^k^
Cl_p_ (L/min∙70 kg)	1.88(5.3%)			1.99(2.5%)
Cl_p_ (L/min∙70 kg)				
of POP_1_		2.52(9.7%)		
of POP_2_		2.13(3.8%)		
Cl_p_ (L/min∙70 kg)				
of POP_bw<d_			15.4(29.3%)	
of POP_bw≥d_			2.03(2.8%)	
*k*				
of POP_1_		1.32(4.2%)		
of POP_2_		0.68(8.9%)		
*k*				
of POP_bw<d_			1.68(8.4%)	
of POP_bw≥d_			0.61(5.9%)	
*π* (i = 1)		0.2(27.7%)		
*d* (kg)			16.5 FIX	
$$ k = {k_0} - {k_{max}} \cdot {\text{b}}{{\text{w}}^\gamma }/({k_{50}}^\gamma + {\text{b}}{{\text{w}}^\gamma }) $$				
*k*_*0*_				1.35 FIX
*k*_*max*_				0.79(8.5%)
*k*_*50*_ (kg)				3.71(7.4%)
γ				5.1 FIX
Q2(L/min)	1.81 (6.2%)	1.82 (6.3%)	1.8 (6.2%)	1.76 (6.6%)
Q3 (L/min)	=Q3_p_⋅(bw/70)	=Q3_p_⋅(bw/70)	=Q3_p_⋅(bw/70)	=Q3_p_⋅(bw/70)
Q3_p_ (L/min∙70 kg)	1.55 (4.8%)	1.54(4.8%)	1.55 (4.8%)	1.59 (5.5%)
V1 (L)	5.18 (10.6%)	4.94 (11.4%)	5.06 (11.1%)	4.9 (11.8%)
V2 (L)	=V2_p_∙(bw/70)	=V2_p_∙(bw/70)	=V2_p_∙(bw/70)	=V2_p_∙(bw/70)
V2_p_ (L/70 kg)	23.9 (10.5%)	23.4 (10.8%)	23.1 (10.7%)	23.9 (10.4%)
V3 (L)	=V3_p_⋅(bw/70)	=V3_p_⋅(bw/70)	=V3_p_⋅(bw/70)	=V3_p_⋅(bw/70)
V3_p_ (L/70 kg)	228 (6.8%)	219 (7.0%)	216 (7.0%)	226 (8.6%)
Inter-individual variability				
ω^2^ CL	0.44(20.7%)	0.11 (17.4%)	0.21 (24.5%)	0.08(17.1%)
ω^2^ V1	1.55(15.2%)	1.52(16.0%)	1.53(15.9%)	1.53(16.3%)
ω^2^ Q3	0.23(14.5%)	0.22(16.3%)	0.22(15.7%)	0.27(20.1%)
ω^2^ V2	0.81(11%)	0.82 (11.4%)	0.81(11.4%)	0.82(11.5%)
ω^2^ V3	0.3(17.4%)	0.31(17.8%)	0.32(17.4%)	0.76(39.3%)
Residual error				
σ^2^ additive	0.06(10.1%)	0.06(10.2%)	0.06(10.3%)	0.06(10.3%)
OFV	−2339.7	−2486.2	−2464.4	−2,489

bw=bodyweight; *π* = the probability of the individuals belonging to *POP1* in the mixture model (Model II); *d* = the cut-point in bodyweight dividing the two sub-populations in the bodyweight-cut-point separated model (Model III); *k*_*0*_= value of the exponent at a theoretical bodyweight of 0 kg; *k*_*max*_= the maximum decrease of the exponent; *k*_*50*_= the bodyweight at which a 50% decrease in the maximum decrease of exponent is attained; γ= the Hill coefficient determining the steepness of sigmoidal decline in the exponent

**Fig. 1:**
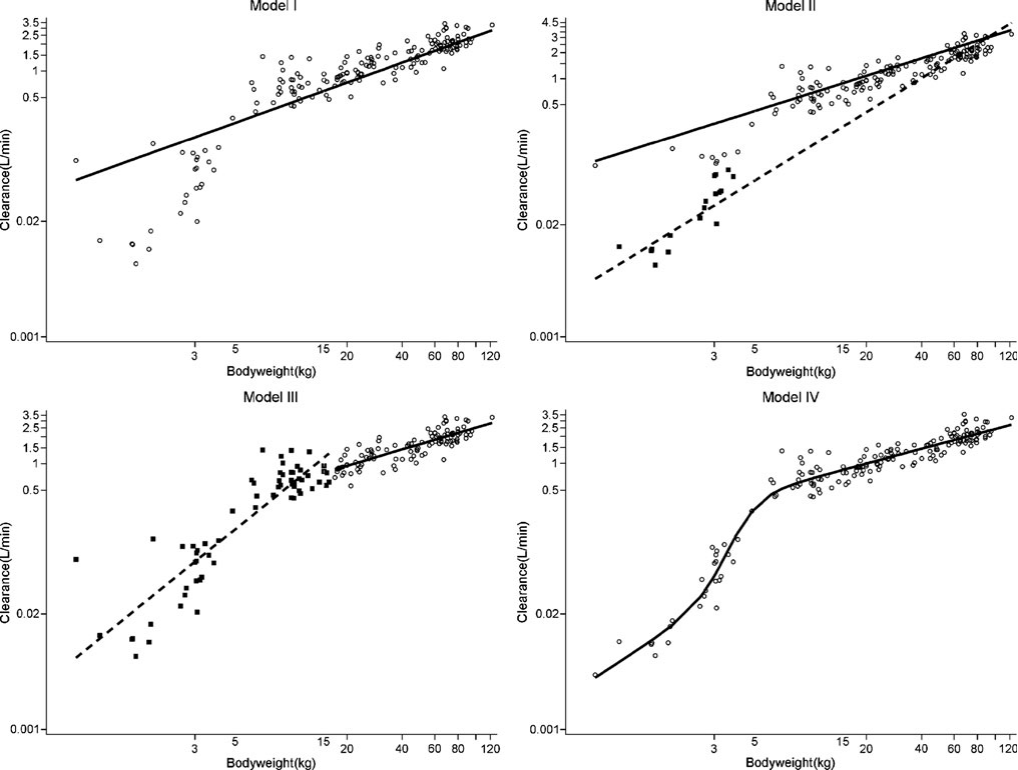
Plots of *post hoc* and population clearance values *versus* bodyweight. For the 3/4 allometric model (Model I), ○: individual *post hoc* clearances, solid line: population predicted clearance curve; the mixture model (Model II), ■: individual *post hoc* clearances of POP_1_, ○: individual *post hoc* clearances of POP_2_, dashed line: population predicted clearance curve of the POP_1_, solid line: population predicted clearance curve of POP_2_; the bodyweight-cut-point separated model (Model III), ■: individual *post hoc* clearances of POP_bw<d_, ○: individual *post hoc* clearances of POP_bw*≥*d_, dashed line: population predicted clearance curve of the POP_bw<d_, solid line: population predicted clearance curve of POP_bw*≥*d_; the bodyweight-dependent exponent (BDE) model (Model IV), ○: individual *post hoc* clearances, solid line: population predicted clearance curve.

The 3/4 allometric model (Model I) with a fixed allometric exponent of 0.75 had an OFV of −2339.7 (Table II). With this model population values of propofol clearance in children weighing less than 15 kg, were both over-estimated (in children between 0.5 and 5 kg) and under-estimated (in children between 5 and 15 kg) (Fig. 1).

For the mixture model (Model II) the OFV decreased significantly (*p* < 0.001) compared to Model I (Table II). The estimated allometric exponent value for clearance of population *POP*_*1*_ was less than 1 (0.676), whereas its value for clearance of population *POP*_*2*_ was greater than 1 (1.321) (Table II). The probability of an individual belonging to *POP*_*1*_ (π) in Model II was found to be 0.2. Figure 1 shows that the population line of Model II described the *post hoc* propofol clearances better than Model I.

The bodyweight-cut-point separated model (Model III) had an OFV of −2464.4, which was an increase of 21.8 (*P* < 0.001) points compared to Model II and a decrease of 124.7 points (*p* < 0.001) compared to Model I (Table II). The bodyweight cut-point was estimated to be 16.5 kg, which was fixed to this value later on to obtain a successful covariance step. The estimated exponents of populations *POP*_*bw<d*_ and *POP*_*bw≥d*_ were 1.68 and 0.614, respectively (Table II). Figure 1 shows that Model III results in improved the fit of the population line to the *post hoc* clearances compared to Model I.

The bodyweight-dependent exponent model (Model IV) had the lowest OFV of −2,489 of the four allometric covariate models, which was significantly superior over the 3/4 allometric model and the bodyweight-cut-point separated model (*p* < 0.001) (Table II). The OFV of this model was not significantly different from the OFV of the mixture model. In order to get a successful covariance step, the exponent of bodyweight at 0 kg *(k*_*0*_) and the hill factor (*γ)* were fixed to the estimated values from a successful run without covariance step, which were 1.35 and 5.24, respectively. Visually, Model IV had the best overall distribution of *post hoc* clearances *versus* population clearance (Fig. 1). Finally, model IV had the lowest inter-individual variability in clearance (Table II), indicating that this new equation indeed accounts for variability in clearance in the entire range of bodyweight of the subjects.

Table III lists the%RMSE values for the different paediatric subpopulations for all four covariate models. The precision of clearance prediction shows a trend of decrease from adult population to neonate population for the four models. In neonates, infants, and children, the bodyweight-dependent exponent model (Model IV) had the lowest%RMSE, and thus the highest precision, of the four models. In toddlers, the mixture model (Model II) had the lowest%RMSE value compared to other models.

**Table III:** %RMSE of the Four Bodyweight-Based Exponential Equations for the Different Human Subpopulations, Model I: 3/4 Allometric Model; Model II: the Mixture Model; Model III: the Bodyweight-Cut-Point Separated Model; Model IV: the Bodyweight-Dependent Exponent (BDE) Model

Population	Model I (%)	Model II (%)	Model III (%)	Model IV (%)
Neonates	160	149	61	49
Infants	63	41	53	40
Toddlers	25	37	38	33
Children	32	26	26	23
Adolescents	24	53	30	27
Adults I & II	24	26	21	20

**Fig. 2:**
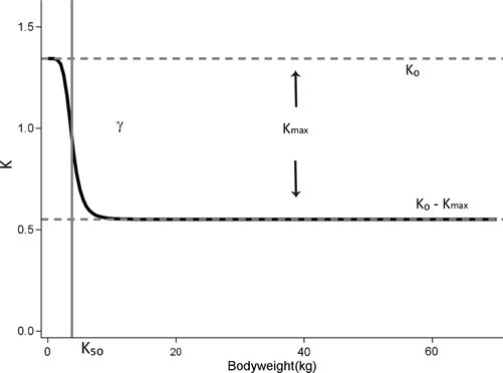
The relationship between the allometric exponent *k* and bodyweight in the bodyweight-dependent exponent (BDE) model for clearance (Model IV, Eq. 5). The parameter *k*_*0*_ represents the value of the exponent at a theoretical bodyweight of 0 kg, *k*_*max*_ represents the maximum decrease of the exponent, *k*_*50*_ represents bodyweight at which 50% of the maximum decrease in the exponent is attained, and *γ* represents the Hill coefficient that determines the steepness of the sigmoidal decline.

**Fig. 3:**
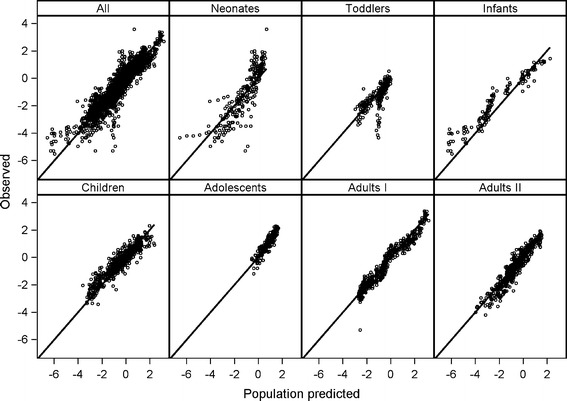
Observed *versus* population predicted propofol concentrations of the bodyweight-dependent exponent model (Model IV). ‘All’ presents data of all datasets together. Other panels represent data of neonates (24), infants (25), toddlers (26), children (27), adolescents (15), adults I (28) and adults II (29).

**Fig. 4:**
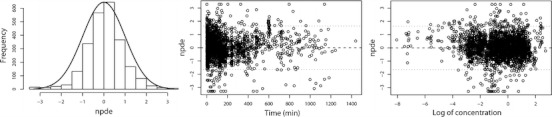
NPDE results of the final PK model for propofol. *Left panel*: histogram of NPDE distribution in the full dataset with the solid line representing a normal distribution as a reference; *Middle panel*: NPDE *versus* time; *Right panel*: NPDE *versus* log transformed concentration.

Based on these results, Model IV was selected as covariate model and further optimized. The parameter estimates of the final PK model are listed in Table IV. Figure 2 shows how the bodyweight dependent exponent for clearance (*k,* Eq. 5) changes with bodyweight according to the estimated parameters of Eq. 5 in Table IV. The figure shows that *k* decreased from a highest value of 1.35 (*k*_*0*_) at the theoretical bodyweight zero to a lowest value of 0.65 (*k*_*0*_-*k*_*max*_). At a bodyweight of 3.78 kg (*k*_*50*_), half of the maximum decrease was reached (Table IV). The Hill coefficient (*γ*) of 5.24 reflected the rapid decrease in the exponent (*k*) with bodyweight (Fig. 2).Concerning other parameters than clearance, a linear function for bodyweight on V1 for the children who were younger than 100 days was identified, while for V2 a power equation based on bodyweight was found. Inclusion of these covariates resulted in a further decrease of 91.7 point (*p* < 0.001) in the OFV compared to Model IV. The observed *versus* population predicted plots stratified by study in Fig. 3 confirm that the final model not only describes the study population as a whole, but also the individual study populations without bias. Results of the bootstrap analysis show that the median estimated values based on resampled data were close to the estimated values from the final model fit of the original data and that all CV percentages are below 50%. This suggests the final model to be stable and the estimated parameter values to be accurate and precise. The results of the bootstrap are of particular relevance for the parameters that were fixed in model IV, i.e. *k*_*0*_ and *γ*, as they justify the actual value these parameters were fixed to. Furthermore, the results from the normalized prediction distribution errors (NPDE) analysis in Fig. 4 show that the model can predict the median concentration in the overall dataset accurately, even though there is a slight over-prediction of the variability in the model. Finally, no trend in the plots of NPDE *versus* time and predicted concentration were observed.

**Table IV:** Parameter Estimates, Bootstrap Results and Their Corresponding Coefficients of Variation (CV%) Values for the Final PK Model

Parameter	Final model	Bootstrap^*a*^
Fixed effect		
Cl (L/min)	= Cl_p_∙(bw/70)^k^	= Cl_p_∙(bw/70)^k^
Cl_p_ (L/min∙70 kg)	2.02(2.6%)	2.02(2.5%)
$$ {\text{k}} = {{\text{k}}_0} - {{\text{k}}_{{ \max }}} \cdot {\text{b}}{{\text{w}}^\gamma }/({{\text{k}}_{{5}0}}^\gamma + {\text{b}}{{\text{w}}^\gamma }) $$		
*k*_*0*_	1.34 FIX	1.35(6.2%)
*k*_*max*_	0.79(12.2%)	0.80(10.7%)
*k*_*50*_ *(kg)*	3.78(15.1%)	3.75(7.5%)
γ	5.24 FIX	5.25(41.6%)
Q2(L/min)	1.77(6.3%)	1.72(7.9%)
Q3 (L/min)	=Q3_p_⋅(bw/70)	=Q3_p_⋅(bw/70)
Q3_p_ (L/min∙70 kg)	1.65(5.0%)	1.64 (4.8%)
V1 (L)	if PNA ≥ 100 then = V1_p_, if PNA < 100 then = V1_p_⋅(bw/70)	
V1_p_ (L)	7.58(12.4%)	7.69(10.1%)
V2 (L)	=V2_p_∙(bw/70)^*m*^	=V2_p_∙(bw/70)^*m*^
V2_p_ (L/70 kg)	15.5(14.7%)	15.7 (11.4%)
*m*	0.55(17.5%)	0.55(10.8%)
V3 (L)	=V3_p_⋅(bw/70)	=V3_p_⋅(bw/70)
V3_p_ (L/70 kg)	221(8.9%)	225(8.9%)
Inter-individual variability		
ω^2^ CL	0.09(18.0%)	0.08(19.4%)
ω^2^ V1	1.19(41.3%)	1.22 (18.3%)
ω^2^ Q3	0.25(17.0%)	0.26(19.9%)
ω^2^ V2	0.52(40.8%)	0.54(16.6%)
ω^2^ V3	0.71(44.0%)	0.75(46%)
Residual error		
σ^2^ additive	0.06(10.3%)	0.06 (10.1%)
OFV	−2580.7	

^*a*^Mean estimated parameter values and their coefficient of variation in percentage from 200 stratified bootstrapping re-samples

bw=bodyweight; *k*_*0*_= the exponent at a theoretical bodyweight of 0 kg; *k*_*max*_= the maximum decrease of the exponent; *k*_*50*_= the bodyweight at which a 50% decrease in the maximum decrease of exponent is attained; γ= the Hill coefficient determining the steepness of sigmoidal decline in the exponent; *m* = the exponent of bodyweight on V2

**Fig. 5:**
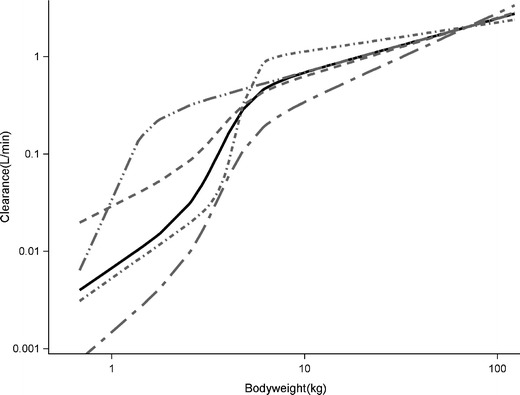
Simulations for the bodyweight-dependent exponent (BDE) model (Model IV) on the basis of different values for the parameters of Model IV (Eq. 5). Solid black curve: BDE curve with *k*_*0*_ = 1.34, *k*_*max*_ = 0.79, *k*_*50*_ =3.78, γ = 5.24 (final PK model); dashed grey curve: BDE curve with *k*_*0*_ = 1, *k*_*max*_ = 0.4, *k*_*50*_ = 3.78, γ = 5; long dash short dash grey curve: BDE curve with *k*_*0*_ = 1.7, *k*_*max*_ = 0.8, *k*_*50*_ = 3.78, γ = 5; short dash dot grey curve: BDE curve with *k*_*0*_ = 1.4, *k*_*max*_ = 1.1, *k*_*50*_ = 4.5, γ = 10; medium dash double dot grey curve: BDE curve with *k*_*0*_ = 1.34, *k*_*max*_ = 0.8, *k*_*50*_ = 1, γ = 5.

Figure 5 shows how variation in the parameters *k*_*0*_, *k*_*max*_, *k*_*50*_, and *γ* of the bodyweight dependent exponent *k* of Eq. 5 results in different curves for clearance *versus* bodyweight, including the observed curve in the current analysis on propofol clearance. This figure illustrates that the developed bodyweight-dependent exponent model is capable of capturing different maturation profiles of clearance *versus* bodyweight allowing in principle to be applied to different drugs and/or different metabolic pathways.

## DISCUSSION

In this study, we tested four different allometric equations to capture changes in propofol clearance in seven datasets comprising almost every stage of human life. We found that fixing the allometric exponent to 0.75 (the 3/4 allometric model, Model I) resulted in adequate description of clearance in adults, adolescents, children and toddlers, but yielded significant under-prediction in infants and over-prediction in term and preterm neonates. Results of the mixture model (Model II) and the bodyweight-cut-point separated model (Model III) showed that an allometric exponent other than 0.75 was more suitable for neonates and infants. In fact, both models identified an allometric exponent greater than 1 for the subpopulation that was composed of mainly neonates and infants resulting in improved description of clearance in these youngest and lightest patient groups in comparison with the 3/4 allometric model. This has been reported before for morphine clearance in children younger than 3 years of age, where an exponent of 1.44 was found to best describe the developmental changes in clearance (22). Similarly, Mahmood reported that the error in the prediction of clearance decreased when the scaling exponent increased from 0.75 towards 1 when studying different drugs in children less than 1 year of age (13). Even though the overall performance improved significantly, the use of two different allometric exponents for different human subpopulations as implemented in Model II and Model III resulted in ambiguous clearance predictions for the individuals on the boundaries of the paediatric subpopulations. In the current study, the observed issue of different exponents at different weight ranges was overcome by implementing an allometric equation with an exponent that varies with bodyweight. This bodyweight-dependent exponent (BDE) model contains a continuous bodyweight-based equation which allowed for the description of maturational changes in propofol clearance in individuals covering the entire human life-span. While the current model needs to be further evaluated in an external validation procedure, according to the advanced model evaluation criteria for pediatric population models (30), the descriptive and predictive performances of this model were affirmed by stratified diagnostics (Fig. 3), bootstrap validation (Table IV), NPDE (Fig. 4), and%RMSE results (Table III).

An important question is whether this bodyweight-dependent exponent may reflect underlying physiological maturation processes. Unfortunately, we were not able to establish a direct link between the change in exponent and the physiological maturation process, due to the lack of physiological information in our data. The results of this study are therefore to be compared to other studies. The reported high value of the exponent at very low bodyweights (Fig. 2), is the result of rapid changes in propofol clearance with bodyweight at these young age ranges (Fig. 1, model IV). The lower value for the exponent at higher weight ranges, results from the accomplishment of the maturation process of propofol clearance (Fig. 2). According to the well-stirred model, hepatic clearance is determined by hepatic extraction ratio and liver blood flow. As propofol is a drug with a high extraction ratio, systemic clearance is driven by liver blood flow (36,37). While there are no good data on hepatic blood flow in relation to age, hepatic blood flow in infants was suggested to be comparable to adult values. Therefore maturation of metabolic capacity could be considered the cause of the rapid changes in clearance in the young individuals (38). For propofol, glucuronidation by the UGT1A9 isoenzyme is the major elimination pathway. UGT1A9 was reported to undergo an age-dependent quantitative differential regulation extending up to 24 months of age (39), and it was found that only part of the reduced glucuronidation rate was compensated by hydroxylation in neonates (40). Even though this cannot be proven, it can be speculated that the rapid change in the bodyweight-dependent exponent at low bodyweight ranges may be the result of the change in the capacity of the UGT1A9 isoenzyme.

In recent years, there has been a debate on how bodyweight and age, which are two correlated covariates in the paediatric population, should be included in population pharmacokinetic models. It has been proposed that the 3/4 allometric equation can be used to standardize drug clearance to the average adult bodyweight of 70 kg, after which age-based equations can be added to account for maturational differences in the younger populations compared to the older ones (1,41). These age based functions are needed because poor prediction performance of the 3/4 allometric model can be expected when scaling clearance to children under a certain age (15–17). However, this addition of age to the 3/4 allometric bodyweight based scaling function may result in collinearity. The effect of the collinearity on parameter estimates in nonlinear mixed effect models has already been studied and it was found that high collinearity between predictors, defined as data collinearity, increased the bias of the parameter estimates and enlarged the corresponding standard errors (20). More recently, it has been shown that when one of two correlated covariates that contain information about a model parameter is pre-selected over the other, the predictive performance of the resulting model may be diminished, unless the pre-selected covariate relationship reflects the true biological relationship (21). With the great amount of theoretical and experimental evidence against the 3/4 allometric model (6–12,14), and the risk of collinearity when introducing age to correct for deviations from this model, new scaling approaches are needed.

The bodyweight-dependent exponent model we described in this paper are of particular relevance for scaling clearance parameters to the youngest age ranges including infants, term and preterm neonates. Since in these age groups changes in the pharmacokinetics may be expected to be large, there is currently great interest in scaling clearance parameters from older populations to neonates or infants younger than 2 years of age (15,16,42). It is however uncertain whether the parameters of the bodyweight-dependent exponent model we developed for propofol can be generalized to other drugs. Propofol has very specific characteristics such as a high extraction ratio, direct glucuronidation and high lipophilicity. However, as Fig. 5 shows, the function is very flexible, and in our opinion the proposed model can be applied to other drugs provided data in young children are available to estimate the exact parameters of Eq. 5 of the bodyweight dependent exponent model for that drug.

## CONCLUSION

In this study, we have developed a model for scaling propofol clearance over the entire human life-span with a single continuous bodyweight based equation, in which the exponent of the equation varies with bodyweight. The flexibility of this bodyweight-dependent exponent (BDE) model may increase the applicability of this type of models to scale clearance of other drugs over large parts of the human life-span. This function may provide an alternative for allometric scaling approaches in the extrapolation of drug clearances from older to younger human age-ranges.

## ABBREVIATIONS

BDE: bodyweight-dependent exponent
EDTA: ethylenediaminetetraacetic acid
NPDE: normalized prediction distribution errors
RMSE: root mean square error
